# A Race-Specific, DNA Methylation Analysis of Aging in Normal Rectum: Implications for the Biology of Aging and Its Relationship to Rectal Cancer

**DOI:** 10.3390/cancers15010045

**Published:** 2022-12-22

**Authors:** Matthew A. Devall, Xiangqing Sun, Stephen Eaton, Gregory S. Cooper, Joseph E. Willis, Daniel J. Weisenberger, Graham Casey, Li Li

**Affiliations:** 1 Department of Family Medicine, University of Virginia, Charlottesville, VA 22903, USA; 2 Department of Medicine, University Hospitals Cleveland Medical Center, Case Western Reserve University, Cleveland, OH 44106, USA; 3 Department of Pathology, Case Western Reserve University/University Hospitals Cleveland Medical Center, Cleveland, OH 44106, USA; 4 Department of Biochemistry and Molecular Medicine, University of Southern California, Los Angeles, CA 90007, USA; 5 Center for Public Health Genomics, University of Virginia, Charlottesville, VA 22908, USA; 6 University of Virginia Comprehensive Cancer Center, University of Virginia, Charlottesville, VA 22908, USA

**Keywords:** rectum, colorectal cancer, DNA methylation, epigenetic aging, racial disparities, African American, aging

## Abstract

**Simple Summary:**

Racial disparities exist within colorectal cancer (CRC) incidence and mortality, with African Americans (AA) exhibiting a greater risk of developing and dying from the disease than European Americans (EA). This disparity is mirrored in scientific research, where an inadequate number of omic-based studies have sought to recruit AA populations, severely limiting the scope of subsequent findings. Aberrant DNA methylation is a hallmark of CRC, with global and region-specific differences being attributed to disease subtype, pathogenesis and survival. However, studies on cancerous tissue are limited in their ability to infer risk mechanisms occurring in healthy cells. Here, we explore the effects of aging, a well-known CRC risk factor, on DNA methylation in normal rectum of AA and EA subjects. Our findings highlight an interplay between race and age that impacts DNA methylation and, potentially, rectal cancer risk.

**Abstract:**

Approximately 90% of colorectal cancer (CRC) develop over the age of 50, highlighting the important role of aging in CRC risk. African Americans (AAs) shoulder a greater CRC burden than European Americans (EA) and are more likely to develop CRC at a younger age. The effects of aging in AA and EA normal rectal tissue have yet to be defined. Here, we performed epigenome-wide DNA methylation analysis in the first, large-scale biracial cohort of normal rectum (*n* = 140 samples). We identified increased epigenetic age acceleration in EA than AA rectum (*p* = 3.91 × 10^−4^) using linear regression. We also identified differentially methylated regions (DMRs) associated with chronological aging in AA and EA, separately using DMRcate. Next, a consensus set of regions associated with cancer was identified through DMR analysis of two rectal cancer cohorts. The vast majority of AA DMRs were present in our analysis of aging in rectum of EA subjects, though rates of epigenetic drift were significantly greater in AA (*p* = 1.94 × 10^−45^). However, 3.66-fold more DMRs were associated with aging in rectum of EA subjects, many of which were also associated with rectal cancer. Our findings reveal a novel relationship between race, age, DNA methylation and rectal cancer risk that warrants further investigation.

## 1. Introduction

Advanced age is a well-established risk factor for colorectal cancer (CRC). Aging adversely affects a variety of biological processes, such as metabolism [1], the immune response [2], genomic instability [3] and telomere shortening [4]. The exact mechanisms underlying these age-related changes are not fully understood, though studies have alluded to a role for alterations to DNA methylation (DNAm), namely a reduction in the stability of epigenetic marks and the development of epigenetic drift [5]. Chronological aging has a strong, widespread effect on DNAm. Research in human blood has shown that approximately half of autosomal CpG sites assayed using the HM450 array exhibit age-related changes during a period from birth to adolescence [6]. However, these widespread alterations occur at biologically programmable events, such as fetal development [7] and puberty [8]. In contrast, some differences are driven by an age-related reduction in the stability of the epigenome across successive cell divisions. In studies of monozygotic twins, DNAm and histone acetylation levels were found to be less similar in older than younger twin pairs [5], a process broadly defined as epigenetic drift [5,9]. For DNAm, this drift can occur through global hypomethylation [10] or region-specific hypermethylation. Age-related DNA hypermethylation occurs frequently in the vicinity of genes related to complex disease [11], and in regions shown to be affected by race [12]. Together, these findings allude to an interesting interplay between, age, race and disease that warrants further investigation.

CRC is an age-related disease, with approximately 90% of cases attributed to individuals 50 years or older [13]. Since the mid-1980’s, African Americans AAs have been consistently reported to shoulder a greater CRC burden than European Americans (EAs) [14,15]. Incidence of later-onset CRC (>50+) in AAs is 18–29% greater than that observed in EAs [16,17]. AAs have also consistently been reported to develop CRC at a younger age than EAs [16]. This is particularly concerning given that early onset rectal cancer (EOCRC) has been associated with poorer survival rates than same-staged tumors developed in older patients [18,19]. However, recent evidence suggests that the rate of increase of EORC is accelerating faster in EA [16,20]. These findings highlight the pressing need for large-scale, diverse population studies that can aid in the interpretation of mechanisms through which RC risk occurs.

Our group previously generated Illumina Infinium MethylationEPIC arrays (herein, EPIC array) on the first epigenome-wide study of normal AA and EA colon biopsies [21]. We found significantly increased epigenetic aging in matched, AA right than left colon, while the inverse was true for EAs [21]. These patterns mirrored the relative rates of sidedness for CRC observed in AA [20,22] and early onset CRC in EA [16]. Further, our differentially methylated position (DMP) analysis of race in right colon revealed a significant enrichment for AA-specific DNA hypermethylation which included many DMPs previously associated with CRC. Such findings show the promise of DNAm studies in normal colorectal tissue as a means to evaluate risk mechanism.

Age-related differences in normal colon overlap CRC-related differences identified in tumor samples [11]. However, most omic studies of CRC have focused on biopsies generated in colon. Some studies have performed genome-wide (HM450 or greater) analysis on rectal tissue [23,24,25,26,27], though the number of normal rectal biopsies sampled within these cohorts are relatively small and the effects of aging have yet to be considered. To better define the role of aging in rectal tissues, we now extend our previous cohort [21] to include rectal biopsies from 140 healthy individuals. We hypothesize that age-related differences in DNAm identified in normal rectal biopsies overlap regions associated with rectal cancer (RC) and provide novel insight into the complex interplay between aging, race and DNAm in the development of RC.

## 2. Materials and Methods

### 2.1. Patient Population

Subjects included in this study were recruited as a part of the Cleveland Colon Screening and Risk Factors Study. This study was conducted from 2012–2018 in metropolitan Cleveland, Ohio and included subjects who were referred for a screening colonoscopy at University Hospitals Cleveland Medical Center (UHCMC). Two affiliated community sites were also recruited to participate in a comparative study of colonoscopy versus stool DNA testing for screening. Subjects were excluded if they met one of the following: (i) younger than 30, or older than 80; (ii) a personal or family history of a cancer syndrome, such as Hereditary Non-polyposis Colorectal Cancer or Familial Adenomatous Polyposis; (iii) a previous diagnosis of colon adenomas; (iv) a history of colon resection; (v) a diagnosis of inflammatory bowel disease; and (vi) a prior or current cancer diagnosis, except for non-melanoma skin cancer. As such, our study participants were considered to be “healthy” with regard to their colorectum, as they were representative of the average population. All consented subjects were asked to complete a collection of stool samples for DNA testing prior to colonoscopy exams. As an option, subjects scheduled for colonoscopy at the UHCMC main campus were asked also to donate normal colorectal tissues. For the present study, only rectal tissues collected were used for analysis. Data generated from a largely overlapping subset of colon samples have previously been published elsewhere [21].

### 2.2. Epidemiological Data Collection

Relevant clinical data were extracted from medical records and pathological reports. Adenoma status was confirmed by pathology reports. Behavioral and lifestyle information was obtained by a Computer-Assisted Personal Interview based on the NCI Colon Cancer Familial Registry Risk Factor Questionnaire [27].

### 2.3. DNA Extraction, Bisulfite Conversion and Array Processing

Genomic DNA was isolated from rectal tissues using the Qiagen DNA Midi Kit (Qiagen, Valencia, CA, USA). Genomic DNA was eluted in 50–100 µL elution buffer. It was then quantified using Nanodrop technology before bisulfite conversion using the Zymo EZ kit (Zymo Research, Irvine, CA, USA) according to the manufacturer’s recommendations. The quantity of bisulfite-converted DNA, as well as the completeness of bisulfite conversion were assessed through the use of a panel of MethyLight-based real-time PCR quality control assays, as described previously [28]. Bisulfite-converted DNAs were used as a substrate for the Illumina EPIC BeadArrays, as recommended by the manufacturer. Whole genome amplification (WGA) of each sample was performed prior to enzymatic fragmentation. Following this, samples were hybridized to an 8-sample BeadArray overnight. Here, WGA-DNA molecules annealed to locus-specific DNA oligomers linked to individual bead types. The oligomer probe designs follow the Infinium I and II chemistries, in which base extension follows hybridization to a locus-specific oligomer. Afterwards, BeadArrays were scanned and data was recorded as iDAT files.

### 2.4. Preprocessing of Data from Normal Rectal Biopsies

Statistical analysis was carried out in R, version 4.2.1 [29]. For analysis of our novel cohort, probes were normalized using the preprocessQuantile() function of the minfi package [30,31], while specifying sample sex. Probes were removed from downstream analysis if: (i) they had a detection *p*-value > 0.01; (ii) were cross-reactive; (iii) contained a SNP at the single base extension or CpG interrogation site at any minor allele frequency (MAF); (iv) were not present in a CpG context; and (v) were present on either sex chromosome. Additional probes were removed if they failed in at least one sample under default parameters of the openSesame() function [32]. A total of 643,898 and 671,662 CpG sites were available for analysis in AA and EA rectum, respectively.

### 2.5. Preprocessing of Data Generated in External Datasets

For analysis of TCGA-READ dataset [25] (*n* = 96), raw IDAT files were downloaded using TCGAbiolinks [33], while E-MTAB-3027 [34] (*n* = 8 pairs) was downloaded from ArrayExpress [35]. Probe removal and data preprocessing were carried out similarly to the main analysis. However, functional normalization was preferred for TCGA-READ and E-MTAB-3027 by using the preprocessFunnorm() function in minfi [30] while specifying sample sex. This method was specifically designed for use in cancer datasets [36].

### 2.6. Statistical Analysis of Data

DMRs were generated on beta values using DMRcate [37]. In an attempt to correct for technical variation on the array, sentrix ID and position were used as adjustment covariates in the champ.runCombat() function of the R package, ChAMP [38,39], while retaining variation attributed to the variable of interest. This approach was performed using logistically transformed data that was subsequently transformed back to beta values following corrections. Cell composition analysis of samples was performed using the HEpiDISH function() of the EpiDISH package [40]. Regression analysis was performed on each score and the trait of interest. Cell scores that were significantly associated with the trait were incorporated into the regression model as adjustment covariates. Sentrix ID was used as the only adjustment covariate for E-MTAB-3027. However, confounding of the variable of interest (tumor versus normal adjacent tissue (NAT)) with sentrix ID and position prevented the use of this approach for TCGA-READ. As such, for TCGA-READ, 81.81% of technical variation on the array was captured by three principal components using the ctrlsva() function of the ENMix package [41]. A comparison of DMRs across variables of interest (age or tumor status) was then carried out by setting lambda = 1000 and minimum CpGs = 7, for all array data. For HM450 data, C was set to 2 (as default). This value was doubled for EPIC array data to account for the increase in probe density on the EPIC array. For the purpose of this study, four regression models were considered:

DNAm ~ sex + polyp + Fib + body mass index + smoking status + age(1)

DNAm ~ sex + polyp + Neutro + B + body mass index + smoking status + age(2)

DNAm ~ sex + age + PC1 + PC2 + PC3 + Eosino + B + status(3)

DNAm ~ pair + status(4)

sex = biological sex; polyp = whether the individual had a polyp at any site along the colorectum; Fib = fibroblast cell score; body mass index (bmi) = log-transformed measure of BMI; smoking status = factor variable (never/former/current); age = continuous trait corresponding to age at sample index; Neutro = neutrophil cell score; B = B cell score; Eosino = eosinophil cell score; status = factor variable (tumor versus NAT); pair = factor variable corresponding to sample identification; NK = natural killer cell score.

Bonferroni correction was applied to the resulting Stouffer’s *p*-values generated in DMRcate. Only DMRs with P_Adjusted_ < 0.05 were deemed statistically significant. For regression models 3 (TCGA-READ) and 4 (E-MTAB-3027), DMRs identified between phenotypes had to exhibit a mean absolute beta difference greater than 5% to be deemed statistically significant. For regression models 1 and 2 (AA and EA rectum, respectively), a minimal beta difference cut off (0.001%) was used, given the continuous nature of the trait. DMRs that were found to be overlapping between analyses were identified using bedR [42]. DMRs identified in each analysis were first ranked by ordering based on significance within each individual dataset. These ranks were then summed and scaled accordingly.

### 2.7. Epigenetic Aging and Mitotic Rate Analysis

For epigenetic aging analysis, age acceleration residuals were derived from normalized beta values as described in Horvath et al. [43]. A multivariate linear regression was performed to measure the association between race and age acceleration residuals while adjusting for smoking status (never/former/current), BMI and biological sex. Previous studies have suggested that changes of mitotic rates may be particularly relevant to cancer [44]. As such, we also performed a similar multivariate regression analysis of mitotic rate using rates calculated by EPITOC2 [44]. As with our analysis of epigenetic aging, smoking status, BMI and biological sex were considered as adjustment covariates for our regression on race.

## 3. Results

### 3.1. Generation of a Bi-Racial Cohort of Normal Rectal Biopsies from AA and EA Populations

Illumina EPIC arrays were performed on 152 samples (Table S1). Median ages for AA and EA were 54 (range = 44–79 years) and 60 (range = 34–80 years), respectively. No difference in age (*p* = 0.12) was observed across the two racial groups. Technical replicates were generated for five samples. Sample preprocessing measures that led to the greatest reduction in mean absolute deviation of beta values between technical replicates were considered for the study (mean absolute deviation = 4.76 × 10^−4^). After sample preprocessing, the technical replicate with the lowest global levels of DNAm was removed from further analysis. Multidimensional scaling plots revealed that the cohort stratified according to self-reported race and was free from obvious outliers (Figure S1). Seven samples were removed from downstream analysis due to missing phenotypic data of interest (BMI and/or smoking status), leaving a final dataset of 95 AAs and 45 EAs. An overview of our workflow is illustrated in Figure S2.

### 3.2. Race-Specific Epigenetic Aging in Normal Rectal Biopsies

**Figure 1:**
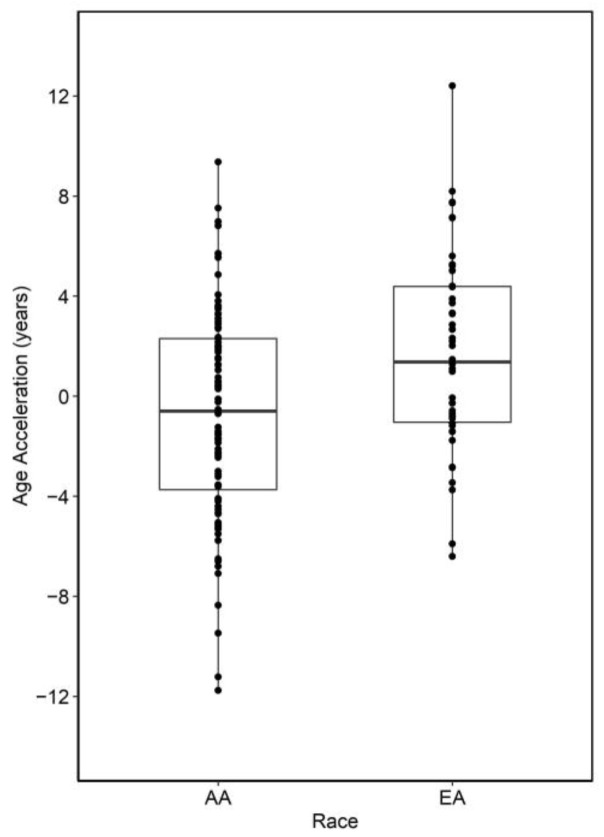
Summary of epigenetic age acceleration analysis between AA and EA normal colon. Positive values indicate samples that are epigenetically older than expected.

We previously observed site and race-specific epigenetic aging in right and left colon from a largely overlapping dataset [21]. Here, we found that accelerated epigenetic aging (Horvath multi-tissue clock) was significantly greater in EA than AA rectum (~2.88 years, *p* = 3.91 × 10^−4^) of a largely overlapping cohort of individuals (Figure 1). A non-significant increase in mitotic rates of EA rectum (*p* = 0.25) was identified in our analysis of Epigenetic Timer of Cancer 2 (EPITOC2) [44].

### 3.3. Race-Specific Patterns of Age-Associated Differential Methylation in Middle-Aged Rectum

**Figure 2:**
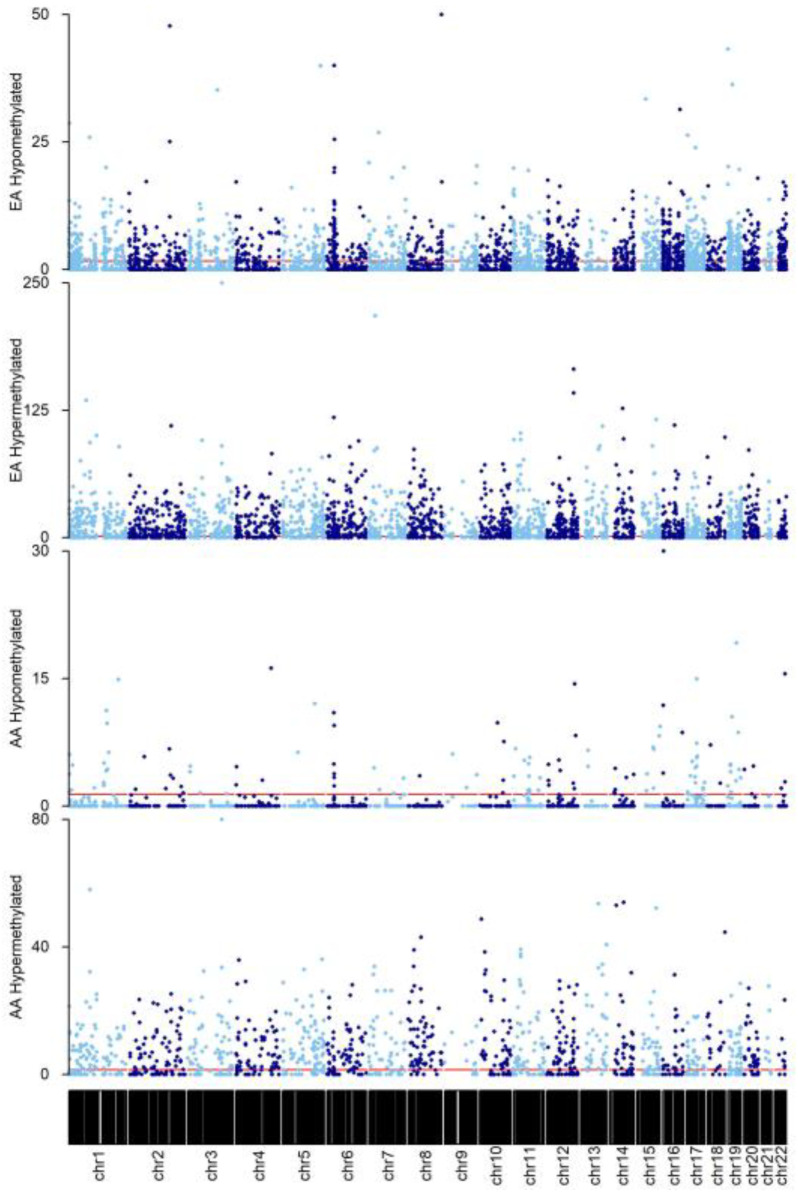
Manhattan plots of DMRs identified in AA and EA rectum. Findings were grouped according to direction of effect for each race. Y-axis represents −log_10_(Bonferroni *p* value). Horizontal red line indicates the cut-off for significance at P_Adjusted_ = 0.05. For visualization purposes, alternating light and blue colors were used to define datapoints in odd and even chromosomes, respectively.

In total, 2955 DMRs were identified in AA rectum (*n* = 95), of which 976 were significantly associated with aging (age-DMRs). DMRcate [37] also led to the identification of 7977 regions of co-methylation in EA rectum (*n* = 45), of which 3575 were age-DMRs (Table S2). Tendencies for direction of effect of significant findings differed between the two racial groups. In EA, 56.53% of age-DMRs were hypermethylated with increasing age, which was in contrast to the 87.00% observed in AA (Figure 2). Of the 976 significant AA age-DMRs, 82.07% (801) overlapped those significant to aging in EA rectum, with all but 11 being concordant for direction of effect. This included DMRs known to be associated with age, such as chr21:31310508–31312687, which corresponded to Glutamate ionotropic receptor kainite type subunit 1 (*GRIK1*) and consists of some of the CpG sites used in the original Horvath clock [43]. A secondary, DMP analysis was performed for the purpose of enrichment testing, as previously [45]. Fisher’s exact test for enrichment revealed a significant overlap between age-DMPs identified in AA and EA rectum (*p* < 2.2 × 10^−16^, Odds Ratio = 3.37).

### 3.4. Epigenetic Drift of Common Age-DMRs Is Greater in AA than EA Rectum

The rate of epigenetic drift was determined by dividing the mean beta difference of each age-DMR by the respective age range of each racial group. The rate of change for consistent age-DMRs was significantly greater in AA than EA rectum (*p* = 1.94 × 10^−45^), with increased rates of epigenetic drift occurring in AA versus EA rectum for 84.34% of common sites. Differences in epigenetic drift occurred at regions corresponding to genes previously associated with CRC. For example, chr18:11751315–11752904 (G protein subunit alpha L (*GNAL*)) drift was greater in EA than AA. *GNAL* hypermethylation has previously been associated with CRC [46]. Conversely, DNA hypermethylation of Integrin subunit alpha 4 (*ITGA4*) has previously been associated with CRC in data generated on peripheral blood mononuclear cells [47], as well as in familial adenomatous polyposis colon organoids [45]. A DMR corresponding to this gene (chr2:182321354–182322898), displayed greater drift in AA than EA.

### 3.5. Contextualization of Age-DMRs in RC

**Figure 3:**
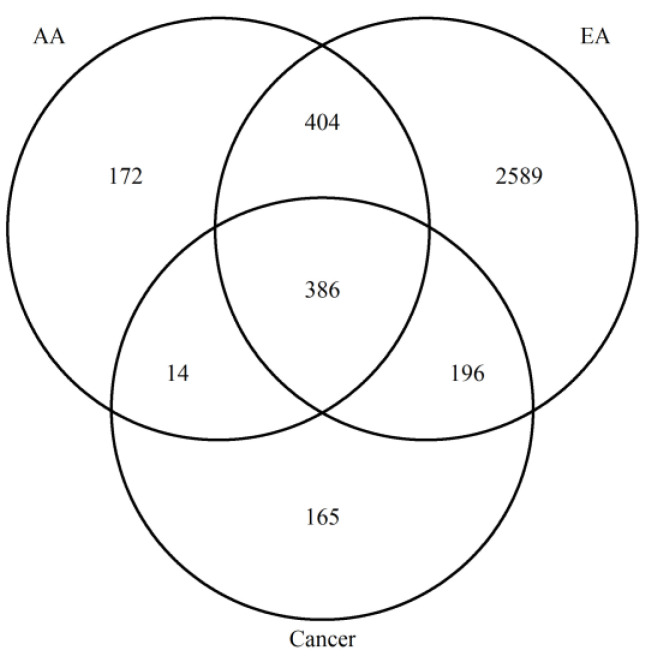
Venn diagram to show the overlap between age- and RC-DMRs identified.

**Figure 4:**
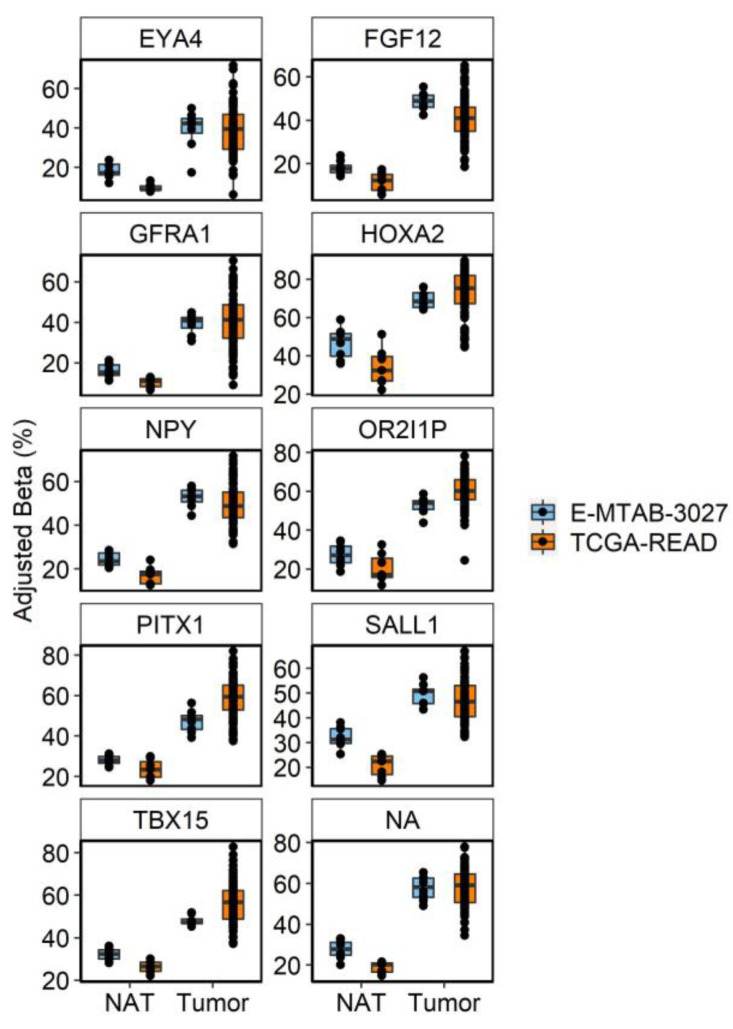
Boxplots of the 10 most significant age-DMRs identified in analysis of RC tumors and NAT in TCGA-READ (orange) and E-MTAB-3027 (blue). Betas were adjusted for technical covariates (sentrix ID and/or sentrix position) prior to plotting using the COMBAT function of the ChAMP package. Individual cytosines within each DMR were averaged on a per sample basis to display the consistency of the average effect of differential methylation at that region across samples.

Our initial findings showed that age-DMRs occurred in regions corresponding to genes that have previously been associated with CRC. To capture the extent by which this occurs, we first identified a set of 761 DMRs consistently associated with RC (RC-DMRs) through a re-analysis of rectal tumors versus NAT in two publicly available datasets [25,34] (Table S2). These 761 DMRs corresponded to 906 distinct genes. Chr6:29520698–29520698 (Olfactory receptor family 2 subfamily I member 1 pseudogene (*OR2I1P*)), was the highest ranked DMR across the two cohorts and was hypermethylated in RC tumors. A total of 596 RC-DMRs were present in at least one analysis of aging, with 571 and 400 overlapping age-DMRs identified in EA and AA, respectively (Table S2; Figure 3). Indeed, 84 of the top 100 ranked RC-DMRs were identified in one, or both, analysis of aging. The vast majority of AA, age-DMRs identified in RC (97%, 388/400), were also age-DMRs in EA. This included eight of the top 10 most significant RC-DMRs (Figure 4). *OR2I1P* and the fifth ranked RC-DMR: chr7:27140797–27143806 (*HOXA2*) were specific to aging in EA rectum. A total of 10 DMRs were also significant for aging in both EA and AA rectum but discordant for direction of effect, of which three were defined as RC-DMRs. Chr4:151502309-151503878 (corresponding to genes including: mab-21 like 2 (*MAB21L2*) and LPS responsive beige-like anchor protein (*LRBA*)), chr14:24803014–24804651 (corresponding to genes including: (*ADCY4*)) and chr17:27044169–27049656 (corresponding to genes including: RAB34, member RAS oncogene family (*RAB34*)) were all hypermethylated in EA rectum with increasing age. Overlapping regions of each of these DMRs were also significantly hypermethylated in both RC analyses. Fisher’s exact test for enrichment revealed that both AA and EA age-DMPs were significantly enriched for RC-DMPs (*p* < 2.20 × 10^−16^), with similar odds ratios for AA (Odds Ratio = 19.03) and EA (Odds Ratio = 17.52). To estimate the novelty of our findings, we compared age-related, RC-DMRs to those found in a previous analysis of colon [11]. A subset of 50 age-related, RC-DMRs had not been previously identified (Table S3). Of these, 39 were specific to EA, six were specific to AA, and five were identified in both analyses. Chr4:151502309–151503878 was among these five common DMRs.

### 3.6. Inferring Functional Effects of Age- and RC-DMRs on Gene Expression

**Figure 5:**
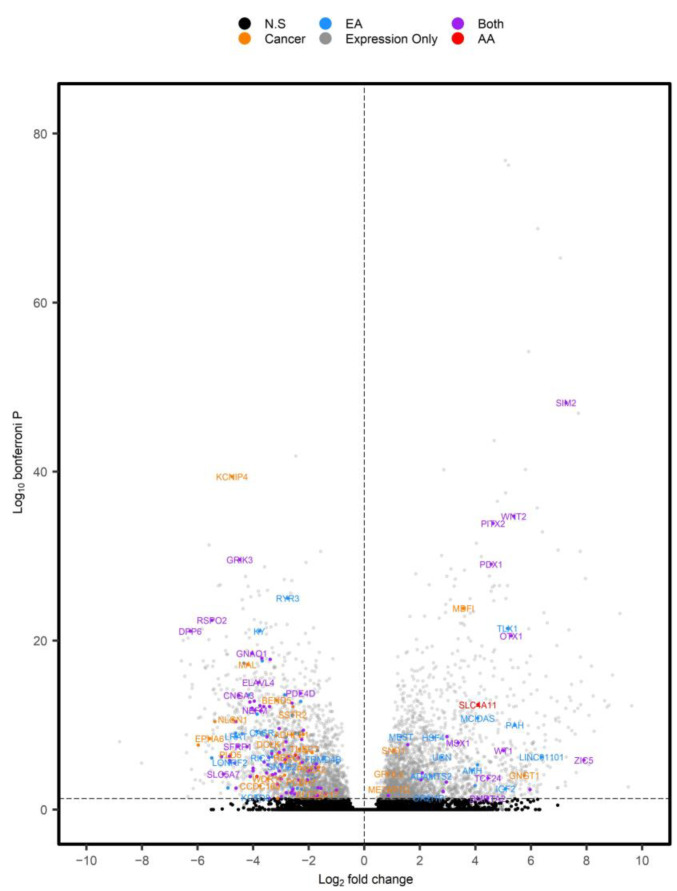
Volcano plot of DEGs identified in TCGA-READ. Positive log2 fold changes represent DEGs overexpressed in tumors versus NAT. Individual genes were color coordinated based on whether they corresponded to RC-DMRs that were associated with aging in AA (red), EA (blue) or both (purple). DEGs (P_Adjusted_ < 0.05) with corresponding RC-DMRs not associated with age are displayed in orange, while significant DEGs not corresponding to a DMR and non-significant (N.S) findings were displayed in grey and black, respectively.

We downloaded and re-analyzed RNA-sequencing data from TCGA-READ (*n* = 164 tumors, *n* = 10 NAT) and related differences in expression to our age-related, RC-DMRs (Figure 5, Table S4). A total of 159 differentially expressed genes (DEGs) were identified that corresponded to an age-related, RC-DMR.

**Figure 6:**
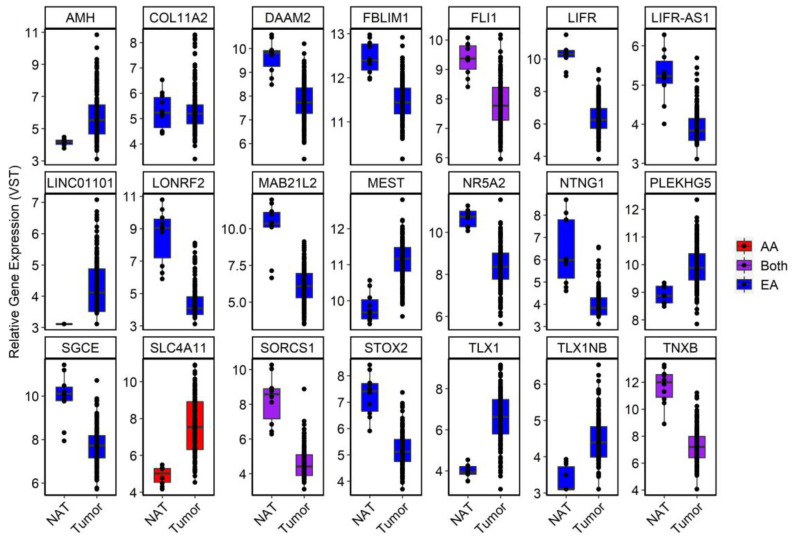
Boxplots of a subset of significant DEGs in TCGA-READ dataset that were deemed novel in our DNAm analysis. For visualization purposes, a variance-stabilizing transformation was applied to each gene using default setting in DESeq2. Genes were coded according to whether the age-DMR was specific to AA (red), EA (blue) or found in both (purple).

Of the 50 novel, age-related, RC-DMRs identified in our analysis, 21 corresponded to significant DEGs identified in expression analysis of TCGA-READ (Figure 6). This included *OR2I1P* (P_Adjusted_ = 0.041) and Solute carrier family 4 member 11 (*SLC4A11*), which were overexpressed in RC (P_Adjusted_ =4.00 × 10^−13^). A DMR corresponding to *SLC4A11* (chr20:31218145–3218905) was among the highest ranked RC-DMRs specific to aging in AA.

Analysis of gene expression aided in relating DMRs to functional effects. For example, the novel DMR at chr4:151502309–151503878 corresponded to *MAB21L2* and *LRBA* (Figure S3). Only *MAB21L2* was found to be significantly reduced in RC tumors (P_Adjusted_ = 2.97 × 10^−13^), with *LRBA* only reaching a nominal trend (P_Nominal_ = 0.09). A highly significant, negative correlation was also observed between this DMR and *MAB21L2* (r = −0.66, *p* = 1.35 × 10^−12^), but not *LRBA*. Chr11:128561007–128565519 (Fli-1 proto-oncogene, ETS transcription factor (*FLI1*) and smooth muscle and endothelial cell enriched migration/differentiation associated lncRNA (*SENCR*)) was the most significant age-DMR identified in EA rectum (P_Adjusted_ = 1.18 × 10^−32^). It was also significant in AA rectum (P_Adjusted_ = 7.01 × 10^−4^) and in RC tumors. This DMR was hypermethylated with increasing age, as well as in RC tumors. *FLI1* was significantly reduced in RC tumors (P_Adjusted_ = 0.017), while *SENCR* was only nominally significant (P*_Nominal_* = 8.48 × 10^−5^), indicating a stronger effect for this DMR at *FLI1* (Figure S4). As expected, this DMR was more significantly correlated to *FLI1* expression (r = −0.44, *p* = 1.43 × 10^−5^) than *SENCR* (r = −0.29, *p* = 5.82 × 10^−3^).

## 4. Discussion

Given the inadequate recruitment of AA individuals in clinical trials and genomic studies, it remains unclear as to whether mechanisms and targets identified within previous studies are applicable to AA [48]. In an attempt to address this disparity, we perform the first, large-scale epigenome wide analysis of normal rectum that places an emphasis on AA recruitment. To define both common and distinct mechanisms through which a well-known CRC risk factor (age) may influence disease risk, we analyzed the effects of aging on DNAm in a cohort of healthy AA and EA rectum, and related findings to those seen in RC tumors. That 84 of the top 100 most significant RC-DMRs were identified in at least one of our analyses of aging provides strong evidence to support our approach for the assessment of CRC risk mechanisms. Through additional analysis of RC tumor expression, we show that many of the age-related, DMRs occur at regions that may drive gene expression differences, thus providing functional insight into these risk-related DMRs exert their effects.

Rising EORC rates in AA and EA are of great concern, as EORC tumors exhibit poorer survival than same-staged tumors developed in older patients [18,19]. Thus, identifying biomarkers and/or mechanism through which perturbed aging is associated with risk is of increasing importance. We identified increased epigenetic age acceleration in normal rectum biopsies of EA versus AA individuals. This may be of clinical interest given that rates of EORC have been rising faster in EA as compared to AA individuals [16]. Questions remain about the functional and biological relevance of epigenetic clocks in CRC [43], driven by mixed associations between CRC risk and epigenetic age acceleration. A previous study in colon biopsies found reduced epigenetic aging in individuals with an increased risk for CRC [49] using PhenoAge [50], with no differences observed in the original Horvath clock [43]. Our research has since found that accelerated epigenetic aging (original Horvath clock) occurs in colon organoids derived from subjects with familial adenomatous polyposis (FAP), who present with a near 100% lifetime risk for CRC, if left untreated [45]. Other clocks have been developed to increase the relevance of findings to cancer, such as EPITOC/EPITOC2 [44]. While we have previously used these methods to identify differences in mitotic rates within the colon that somewhat replicated our Horvath clock analysis, we were unable to replicate these findings here. The reasons for this are unclear. However, our Horvath clock finding strikingly mirrors that which we previously identified [21], that race-specific patterns of epigenetic aging reflect CRC disparities in a manner that appears to be heavily influenced by colorectal location. Thus, our finding adds weight to our hypothesis that the Horvath clock may be a potential biomarker for CRC disease risk.

While useful, the Horvath clock only considers differences observed at a few hundred cytosines. However, epigenetic drift is a stochastic, epigenome-wide process, whereby heritable states of DNAm gradually shift from baseline over an ill-defined period of successive cell divisions. Here, we identified numerous mechanisms through which the process of aging may confer increased risk to RC. The majority of AA, age-DMRs were identified in our analysis of aging in EA rectum. However, common age-DMRs displayed significantly greater epigenetic drift rates in AA than EA rectum. Many of these common regions were also found to be overlapping DMRs associated with RC and displayed aberrant gene expression profiles in RC tumors.

The mechanisms driving this preponderance for increased drift in AAs are unclear, but could be due to a combination of genetic and environmental factors. While epigenetic drift was greater among common age-DMRs, more age-DMRs were identified in EA than AA rectum, and more of these were also identified in RC tumors. For example, *OR2I1P* was the highest ranked RC-DMR. *OR2I1P* was both hypermethylated and overexpressed in RC tumors. The role of this pseudogene in RC is unclear. While *OR2I1P* was previously identified by Joo et al., (2021), their study did not consider the effects of race and our findings allude to a role of this DMR that is restricted to EA rectum. We also identified a subset of novel age-related, RC-DMRs, including 39 that were specific to EA rectum. Of these 50, a DMR corresponding to *FLI1* and *SENCR* was hypermethylated in both EA and AA rectum, though only *FLI1* survived multiple testing corrections in our expression analysis of RC tumors. Negative correlations between DNA methylation and gene expression of *FLI1* have previously been reported in CRC lines, where gene silencing experiments have alluded to potential tumor suppressor functions [51]. Of interest, this DMR was one of the few common age-DMRs where rates of epigenetic drift were greater in EA than AA. A small subset of age-DMRs were also found to be discordant for direction of effect in our AA and EA analysis. Chr4:151502309–151503878 was hypermethylated with increasing age in EA rectum, as well as in RC tumors versus NAT. However, an overlapping region was hypomethylated in AA rectum. Expression of *MAB21L2* was reduced in RC tumors versus NAT. Increased expression of this gene has previously been associated with tumor dormancy in CRC, a state which confers chemotherapy resistance [52].

It is unclear whether the age-related differences in direction of effect lead to altered patterns of gene expression in normal rectal tissue, for which expression analysis of matched rectum is required. It is also unclear what poses the greater RC risk: the rate of epigenetic drift, or the number of RC-related genes affected through the process. Further investigation into such mechanisms is important to better understand the role of aging in this disease.

There are a few limitations in our study. The use of relatively small RC datasets may lead to spurious associations between DMRs and RC status. To overcome this, we only considered consistent findings reported in both cohorts as likely RC-DMRs. However, while the Illumina EPIC array does provide good coverage of the human genome, a greater extent of overlap may be seen in analysis of DNA methylation using more comprehensive, but expensive, technologies such as whole genome bisulfite sequencing. Similarly, while we present the first analysis of aging in AA and EA rectum, larger datasets should be prioritized. These datasets should consider wider age ranges and multiple within-individual samplings. However, that most of our findings were consistent between EA and AA populations suggests that our model accurately captured the molecular mechanisms of aging in otherwise healthy rectum. Future research should aim to determine whether aberrant levels at these sites correspond to changes in gene expression and ultimately, to increased RC risk. Finally, while DNA methylation is a major regulator of gene expression, a comprehensive analysis of other omic layers found to be altered in CRC, genetic variability, lncRNA and microRNAs [53] may further aid in interpreting the biological complexity of processes governing gene expression.

## 5. Conclusions

Our analysis supports the notion that epigenetic age acceleration may be an important biomarker for identifying at-risk individuals for CRC. Further, we found that the process of aging leads to a largely conserved set of DMRs across racial groups. However, age- and RC-DMRs were reported in both AA and EA rectum, highlighting potential targets for more personalized medicine. Future analysis should prioritize determining a functional role of these DMRs, including their role in the modulation of gene expression and how that may contribute to RC risk.

## Data Availability

All data has been made available to Gene Expression Omnibus and can be accessed through accession number: GSE216024.

## Supplementary Materials

The following are available online at https://www.mdpi.com/article/10.3390/cancers15010045/s1, Table S1: Summary of patient demographics used in our analysis. Table S2: Summary of age- and rectal cancer-associated DMRs. Positive mean difference values represent a DMR that was hypermethylated in rectal tumors or is positively correlated with aging. Results were sorted by a rank significance value for aging. DMRs that were found to be overlapping in both analysis and concordant for direction of effect were first ranked individually based on their significance within that analysis. Only DMRs that were found to be significant in both rectal cancer cohorts and concordant for direction of effect were considered. Some, large DMRs overlapped more than one DMR in analysis of another cohort. In these cases, results for the smaller sized DMR were duplicated in the row below. For completeness, all DMRs were presented from each analysis. DMRs that did not show a level of replication in another racial group were indicated by an “NA”. Table S3: Summary of novel, age- and rectal cancer-associated DMRs. Positive mean difference values represent a DMR that was hypermethylated in rectal tumors or is positively correlated with increasing age. Only DMRs that were found to be significant in both rectal cancer cohorts and concordant for direction of effect were considered. DMRs were ranked according to rank significance measures of RC tumor analysis. Some, larger DMRs overlapped more than one DMR in analysis of another cohort. In these cases, results for the smaller sized DMR were duplicated in the row below. For completeness, all DMRs were presented from each analysis. DMRs that did not show a level of replication in another racial group were indicated by an “NA”. Table S4: Summary of differential expression analysis performed on TCGA-READ. Increased log2fold changes correspond to genes found to display increased gene expression in RC versus NAT. Figure S1: MDS plot of the top 2500 most variable single cytosines in normal rectum. Figure S2: Workflow of analysis presented within the current study. Figure S3: Scatterplot demonstrating the correlation between DNA methylation levels at chr4:151502309-151503878 and gene expression levels *of MAB21L2* and *LRBA* in TCGA-READ. Figure S4: Scatterplot demonstrating the correlation between DNA methylation levels at chr11:128561007–128565519 and gene expression levels *of FLI1* and *SNCR* in TCGA-READ.
