# Supplementary material for: A Race-Specific, DNA Methylation Analysis of Aging in Normal Rectum: Implications for the Biology of Aging and Its Relationship to Rectal Cancer
Source: Cancers (Basel). 2022 Dec 22;15(1):45. doi: 10.3390/cancers15010045 (PMC9817986; doi:10.3390/cancers15010045)
Supplement: Supplementary file 1 [file cancers-15-00045-s001.zip › cancers-2015469-SI/SupplementaryFigures.pdf]

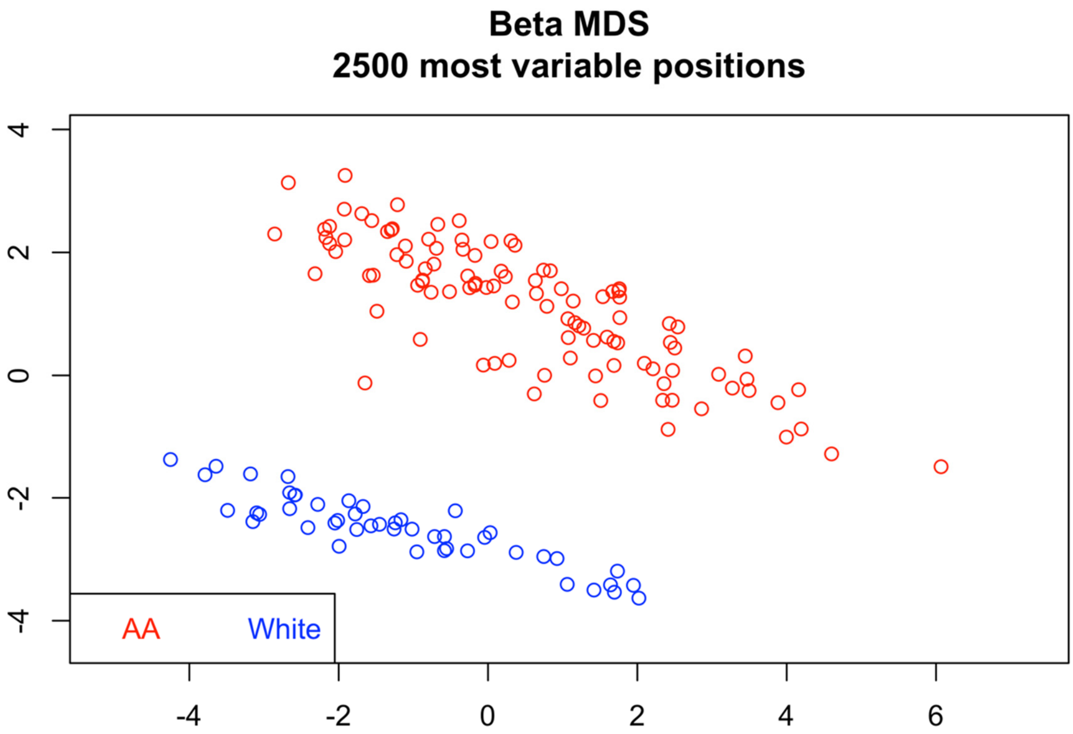

Figure S1: MDS plot of the top 2,500 most variable single cytosines in normal rectum.

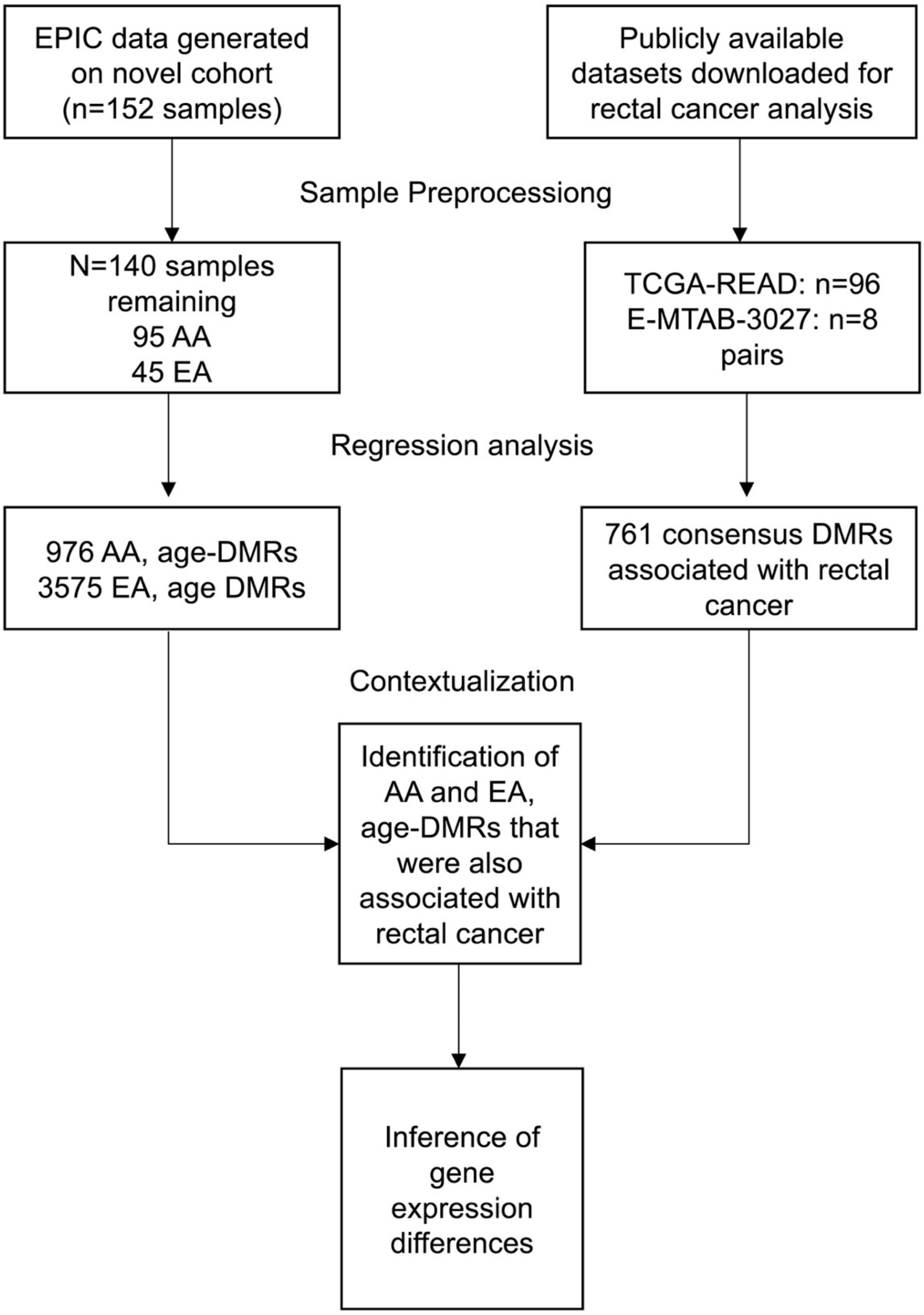

Figure S2: Flow diagram depicting the workflow of analysis presented within the current study.

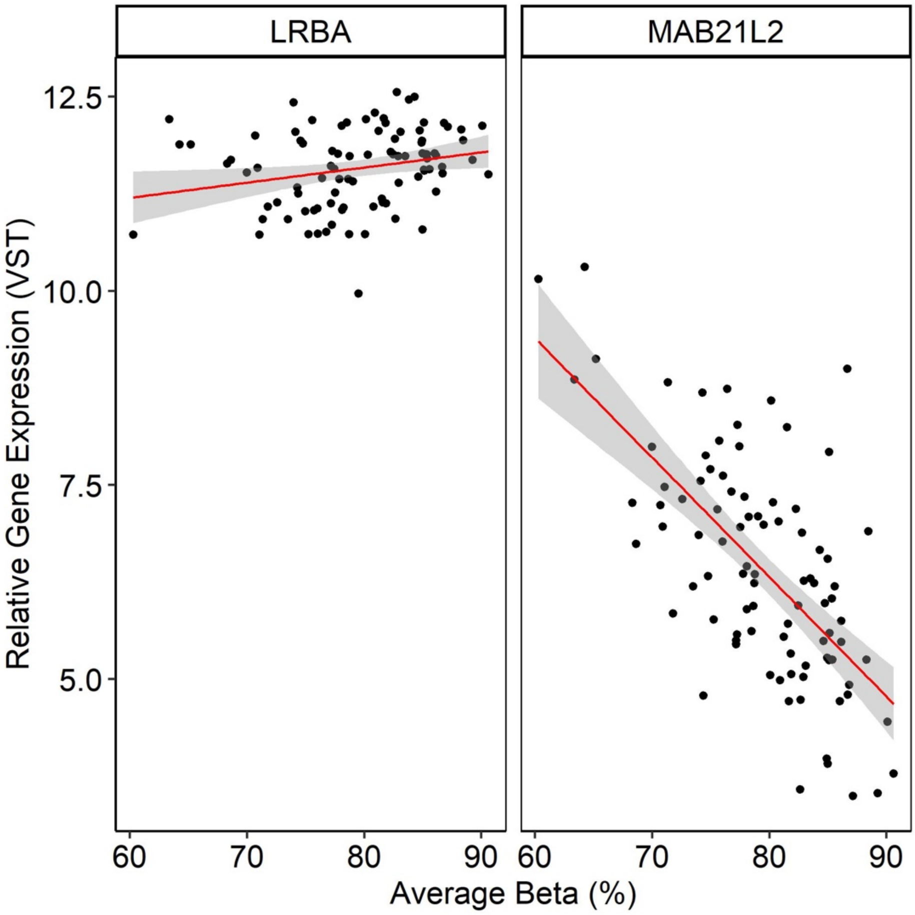

Figure S3: Scatterplot demonstrating the correlation between DNA methylation levels at chr4:151502309-151503878 and gene expression levels *of MAB21L2* and *LRBA* in TCGA-READ.

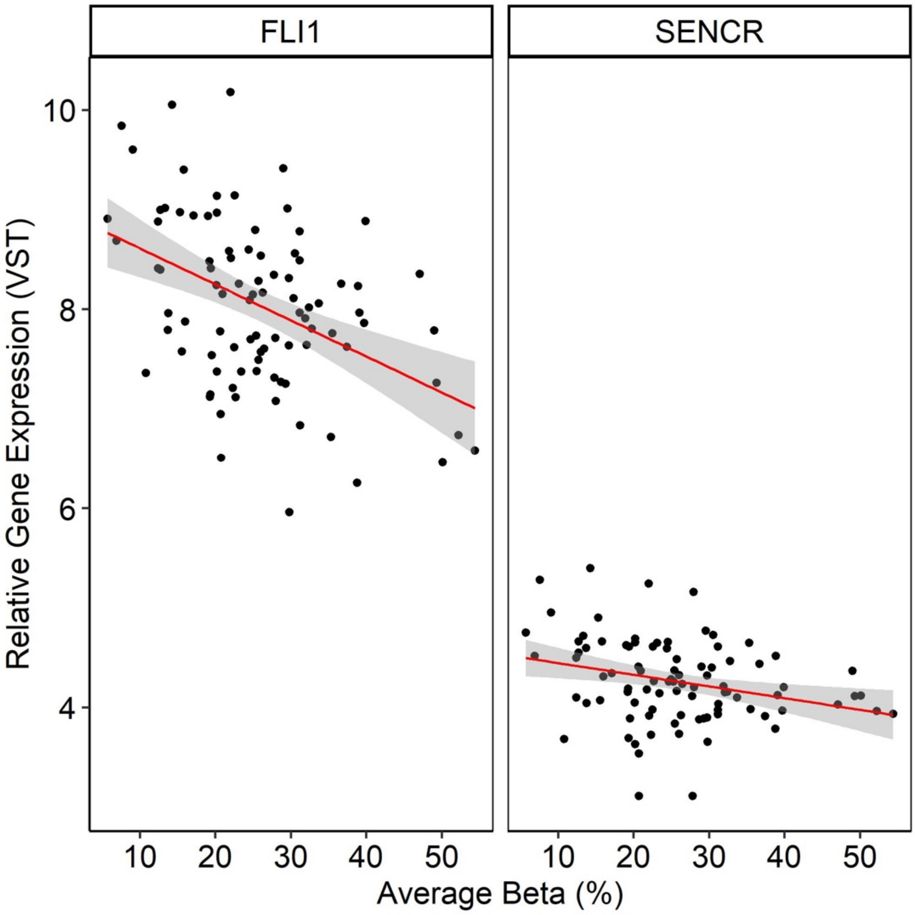

Figure S4: Scatterplot demonstrating the correlation between DNA methylation levels at chr11:128561007-128565519 and gene expression levels *of FLI1* and *SNCR* in TCGA-READ.
